# Facile Preparation of Porous Carbon Derived from Pomelo Peel for Efficient Adsorption of Methylene Blue

**DOI:** 10.3390/molecules27103096

**Published:** 2022-05-11

**Authors:** Wenlin Zhang, Mingwan Liu, Yuhong Zhao, Qinhong Liao

**Affiliations:** 1 Chongqing Key Laboratory of Economic Plant Biotechnology, College of Landscape Architecture and Life Science (Institute of Special Plants), Chongqing University of Arts and Sciences, Yongchuan, Chongqing 402160, China; zhangwenlin88519@126.com (W.Z.); chocolate11702@126.com (M.L.); 2 College of Food Science, Southwest University, Beibei, Chongqing 400716, China; 3 College of Biology and Food Engineering, Chongqing Three Gorges University, Wanzhou, Chongqing 404199, China; zyh0101hyz@163.com

**Keywords:** porous carbon, pomelo peel, adsorbent, methylene blue

## Abstract

Pomelo peel waste-derived porous carbon (PPPC) was prepared by a facile one-step ZnCl_2_ activation method. The preparation parameters of PPPC were the mass ratio of ZnCl_2_ to pomelo peel of 2:1, carbonization temperature of 500 °C, and carbonization time of 1 h. This obtained PPPC possessed abundant macro-,meso-, and micro-porous structures, and a large specific surface area of 939.4 m^2^ g^−1^. Surprisingly, it had excellent adsorption ability for methylene blue, including a high adsorption capacity of 602.4 mg g^−1^ and good reusability. The adsorption isotherm and kinetic fitted with Langmuir and pseudo-second order kinetic models. This work provides a novel strategy for pomelo peel waste utilization and a potential adsorbent for treating dye wastewater.

## 1. Introduction

Organic dye wastewater produced from textile, printing, leather, and paper industries becomes a serious pollution problem once it is discharged into the environment without effective treatment [1]. Methylene blue (MB) is a commonly used dye in these industries and has potential toxicities for aquatic environments and humans [2,3]. Importantly, MB molecule in wastewater is difficult to decompose under natural conditions [4]. Therefore, removing MB from wastewater is urgently necessary.

According to previous reports, many techniques such as adsorption, membrane filtration, biodegradation, photocatalysis, chemical oxidation, and so on, have been used to treat dye wastewater [5]. Among these methods, adsorption is a competitive approach owing to its low cost and easy operation [6]. Porous carbon has many applications in wastewater treatment, electrochemical uses, and medicine [7,8]. Particularly, it is a very promising adsorbent for organic dye removal, attributed to its highly specific surface area and abundant hierarchical pore structures [9]. Previous studies reported that the macropores in porous carbon could promote the mass transfer process, and the mesopores and micropores could catch MB molecules [10,11]. Recently, several porous carbons derived from various biomass raw materials such as ficus carica bast, coconut leaves, and palm shell were developed as adsorbents for MB removal [12,13,14]. However, it is still necessary to seek abundant and low-cost biomass sources to prepare porous carbons with good adsorption performance.

**Figure 1:**
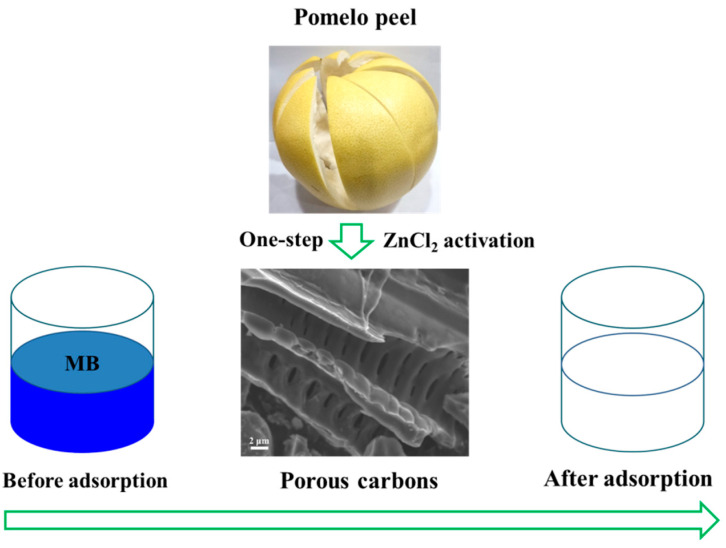
Illustration of the synthesis and adsorption application of PPPC.

Pomelo is widely planted in the south of China and is consumed freshly or processed in large amounts annually. Pomelo peel (PP) accounts for half of fresh fruit weight, which is mostly discarded as waste in juice or jam-processing industries [15]. Seriously, it can cause a series of environmental problems and the wastage of pomelo resources. In addition, PP has a sponge-like mesh porous structure and contains abundant cellulose (46.22%) and hemicellulose (18.84%) [16]. Therefore, using PP as a raw material to prepare porous carbon material is worth considering. Liu et al. prepared PP-derived porous carbon with a BET-specific surface area of 832 m^2^ g^−1^ by the combination of hydrothermal carbonization and KOH activation at 700 °C for 2 h [17]. Li et al. synthesized porous carbon by the pre-carbonization of PP at 450 °C and followed by KOH activation and carbonization at 800 °C [18]. Although its BET-specific surface area can reach up to 1892.10 m^2^ g^−1^, the carbonization time (t) is up to 6 h. It can be found that the above steps are tedious, time consuming, and involve high energy consumption. Moreover, KOH could cause serious corrosion to the synthesis equipment. Herein, PP-derived porous carbon (PPPC) was developed by a one-step ZnCl_2_ activation method (Figure 1), and their adsorption ability and reusability for MB was evaluated.

## 2. Materials and Methods

### 2.1. Materials

The mature ‘Liangpingyou’ pomelos were obtained from a pomelo orchard in Liangping District, Chongqing, China, and their peels were subsequently dried, smashed, and sieved through a 60-mesh sieve. MB were purchased from Sigma-Aldrich Co. LLC. (St Louis, MO, USA). Other chemicals of analytical reagent grade were obtained from Chengdu Kelong Chemical Reagent Co. (Chengdu, Sichuan, China). Ultrapure water was used for all experiments.

### 2.2. Preparation of PPPC

In a typical process, 1.0 g PP powder and 0.5–4 g ZnCl_2_ were added into 50 mL water under vigorous stirring for 2 h. Subsequently, the mixtures were freeze-dried. The dried mixtures were transferred in a tube furnace and heated at 300–700 °C under nitrogen flow with a rate of 5 °C min^−1^ for 0.5–2.5 h. Then, the prepared samples were washed with 1 mol L^−1^ HCl solution and water, respectively. Finally, the products were dried at 60 °C in an oven.

### 2.3. Material Characterizations

The morphology of PPPC was observed by a JEOL JSM-7100F field-emission scanning electron microscope (FESEM, Tokyo, Japan) operated at 10 kV. Nitrogen adsorption-desorption isotherm was measured using a Quadrasorb instrument (Quantachrome, Boynton Beach, FL, USA) at 150 °C, and the data analysis was performed with Quantachrome software. X-ray diffraction (XRD) patterns were studied using a Rigaku Ultima IV X-ray diffractometer with Cu-Kα radiation (λ = 0.15418 nm). Fourier transform-infrared (FTIR) spectrum was recorded using a Nicolet 6700 spectrophotometer (Thermo Fisher, Cleveland, OH, USA). Raman spectrum was performed by a DXR Raman spectroscopy system (Thermo Fisher, Cleveland, OH, USA). Zeta potential was determined using a Malvern Zetasizer Nano ZS 90 spectrometer.

### 2.4. Adsorption and Regeneration Experiments

Adsorption experiments were performed in glass bottles containing 5.0 mg PPPC and 10 mL MB aqueous solutions with various initial concentrations. Subsequently, the mixtures were shaken at 200 rpm with different pH, time, and temperature, respectively. Then, the samples were centrifuged and the supernatant concentrations were determined by a UV-Vis spectrophotometer at 664 nm. For the effect of pH on the adsorption, the different calibration curves were used to determine MB concentration at different pH. The equations of MB adsorption capacity, pseudo-first-order kinetic, pseudo-second-order kinetic, Langmuir, and Freundlich models were listed as follows.

(1) $$q_{e}=\frac{\left(c_{0}{\;-\;c}_{e}\right)V}{m}$$

(2) $$\log\left(q_{e}{\;-\;q}_{t}\right){=\log q}_{e}\;-\;\frac{k_{1}t}{2.303}$$

(3) $$\frac{t}{q_{t}}=\frac{1}{k_{2}q_{e}^{2}}+\frac{t}{q_{e}}$$

(4) $$\frac{c_{e}}{q_{e}}=\frac{c_{e}}{q_{m}}+\frac{1}{{bq}_{m}}$$

(5) $${\log q}_{e}=\log k+\frac{1}{n}{\log c}_{e}$$

where, *q*_e_ (mg g^−1^) is the equilibrium adsorption capacity; *c*_0_ (mg L^−1^) and *c*_e_ (mg L^−1^) are the initial and equilibrium concentrations of MB, respectively; *V* (L) is solution volume; *m* (g) is the mass of PPPC; *q*_t_ (mg g^−1^) is the adsorption capacity at any time; *k*_1_(min^−1^) and *k*_2_ (g mg^−1^ min^−1^) are the kinetics adsorption rate constants; *t* is contact time (min); *q*_m_ (mg g^−1^) is the maximum adsorption capacity; *b* (L mg^−1^*)* is the adsorption constant; *k* is the indicator of adsorption capacity; and 1/*n* is the heterogeneity factor.

For the regeneration study, 5.0 mg of PPPC was added to 10 mL MB solution (100 mg L^−1^) at pH 12 and the mixtures were shaken at 200 rpm for 2 h at 25 °C. After adsorption and centrifugation, the supernatant was discarded leaving PPPC. Then, MB-adsorbed PPPC was added to 10 mL pH = 2 ethanol and shaken at 200 rpm for 10 min. Subsequently, PPPC was isolated from the solution by centrifugation and used for the next cycle. The final concentration of MB was determined by UV-vis spectra. The adsorption-desorption processes were conducted five times.

## 3. Results and Discussion

### 3.1. Synthesis of PPPC

**Figure 2:**
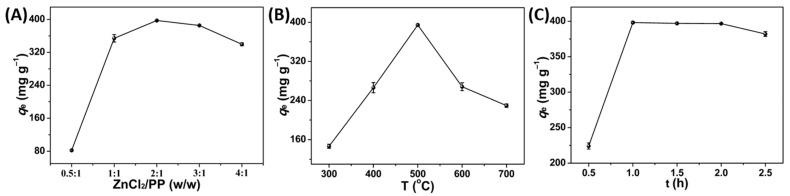
Effect of (**A**) MRZP (T: 500 °C, t: 1 h), (**B**) T (MRZP: 2:1, t: 1 h), and (**C**) t (MRZP: 2:1, T: 500 °C) for MB adsorption on PPPC (pH: 12, *c*_0_: 200 g L^−1^, 2 h, 25 °C).

In the study, PPPC was successfully prepared by a one-step ZnCl_2_ activation method. To obtain PPPC with better adsorption performance for MB, the mass ratio of ZnCl_2_ to PP (MRZP), carbonization temperature (T), and t were optimized by one-factor-at-a-time approach (OFAT). As shown in Figure 2A, *q*_e_ of PPPC increased with the increasing MRZP until 2:1, and reached the highest of 397.4 mg g^−1^ for 2:1. Subsequently, it decreased when MRZP was over 2:1. According to previous reports, it was considered that impregnation with suitable ZnCl_2_ could make PP undergo oxidative degradation and catalytic dehydration during the carbonization process, which led to aromatization and charring of carbon skeleton and formed more pores to improve the *q*_e_ [19]. In addition, *q*_e_ of PPPC increased sharply with the increasing T from 300 °C to 500 °C and reached a maximum of 394.4 mg g^−1^ at 500 °C. However, it decreased when T was over 500 °C (Figure 2B). This was probably because the higher temperature (600 and 700 °C) could destroy the pore structures of PPPC and caused the decrease of specific surface area [17], which led to the decrease of *q*_e_. Moreover, with the extension of t until 1 h, *q*_e_ of PPPC increased gradually (Figure 2C) and reached the highest value of 398.18 mg g^−1^ at 1 h. Then, *q*_e_ showed a decreasing trend over 1 h. It was found that appropriate MRZP, T, and t could improve the adsorption performance of PPPC greatly. Therefore, MRZP of 2:1, T of 500 °C, and t of 1 h were chosen as the optimum preparation parameters of PPPC.

### 3.2. Characterizations of PPPC

**Figure 3:**
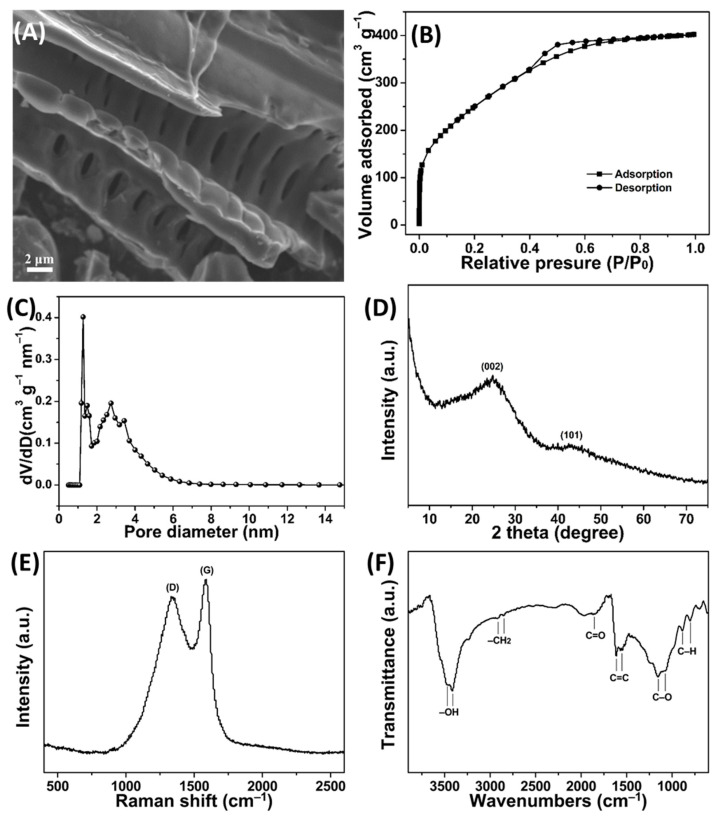
(**A**) SEM image, (**B**) nitrogen adsorption–desorption isotherm, (**C**) pore size distribution, (**D**) XRD pattern, (**E**) Raman, and (**F**) FTIR spectra of PPPC.

FESEM image in Figure 3A showed that the optimized PPPC possessed rich and uniform macropores. To further investigate the mesopore and micropore characteristics as well as the specific surface area of PPPC, the nitrogen adsorption-desorption isotherm was performed. As seen in Figure 3B, the isotherm increased sharply at a low relative pressure of P/P_0_ < 0.05, suggesting that PPPC had abundant micropores [20]. Subsequently, a hysteresis loop appeared on the isotherm of PPPC at 0.4 < P/P_0_ < 1.0, indicating the existence of many mesopores in PPPC [20]. Moreover, the pore size distribution result also proved that PPPC possessed rich micropores and mesopores (Figure 3C). The total pore volume and average pore diameter of PPPC were 0.62 cm^3^ g^−1^ and 2.6 nm. Together with SEM analysis, PPPC indeed had abundant porous structures. Notably, these porous structures not only make MB molecules enter PPPC surface quickly, but also entrap and adsorb more MB molecules to improve the adsorption capacity of PPPC [11]. Particularly, the specific surface area of PPPC was up to 939.4.2 m^2^ g^−^^1^, and it was larger than those of previously reported porous carbons (Table S1), except the KOH-activated PP-based porous carbon (1892.1 m^2^ g^−1^).

XRD pattern was employed for exploring the structure and phase composition of PPPC. The broad two peaks at 25.4° and 43.3° corresponded to the (002) and (101) planes of the disordered carbon layer (Figure 3D), indicating that the carbon with a turbostratic structure with low crystallinity existed in PPPC [21]. According to the previous report, the turbostratic structures were beneficial for the adsorption of dye molecules [22]. Moreover, the low angle region (2θ < 15°) appeared to have high intensity, which was ascribed to the abundant micropores in PPPC [11,21], which confirmed the results of nitrogen adsorption-desorption isotherm and size distribution studies. Raman spectrum showed that the peaks at 1586 cm^−1^ (G-band) and 1334 cm^−1^ (D-band) corresponded to the ordered graphitic carbon and lattice defects carbon in PPPC [23] (Figure 3E), respectively. Furthermore, the *I*_D_/*I*_G_ ratio was calculated to be 0.89, revealing the existence of rich disordered carbons in PPPC. In addition, the full width at half maximum (FWHM) of the D band was obviously wider than that of the G band, indicating a high percentage of disorder and defects in the carbons [21], which was in agreement with the XRD result. FTIR spectrum was used to study the surface organic groups of PPPC. As shown in Figure 3F, the bands at 3458 and 3315 cm^−1^; 2923 and 2854 cm^−1^; 1869 cm^−1^; 1614 and 1567 cm^−1^; 1153 and 1074 cm^−1^; and 889 and 802 cm^−1^, represented –OH, –CH_2_, C=O, C=C, C–O, and C-H groups respectively, which were derived from the carbonization of PP during the carbonization process [3,20]. Based on the above results, it was known that many oxygenated groups existed on the surface of PPPC. To be emphasized, these oxygenated groups were critical for MB adsorption due to the interactions and offered numerous adsorption sites [13].

### 3.3. Adsorption Performance of PPPC

**Figure 4:**
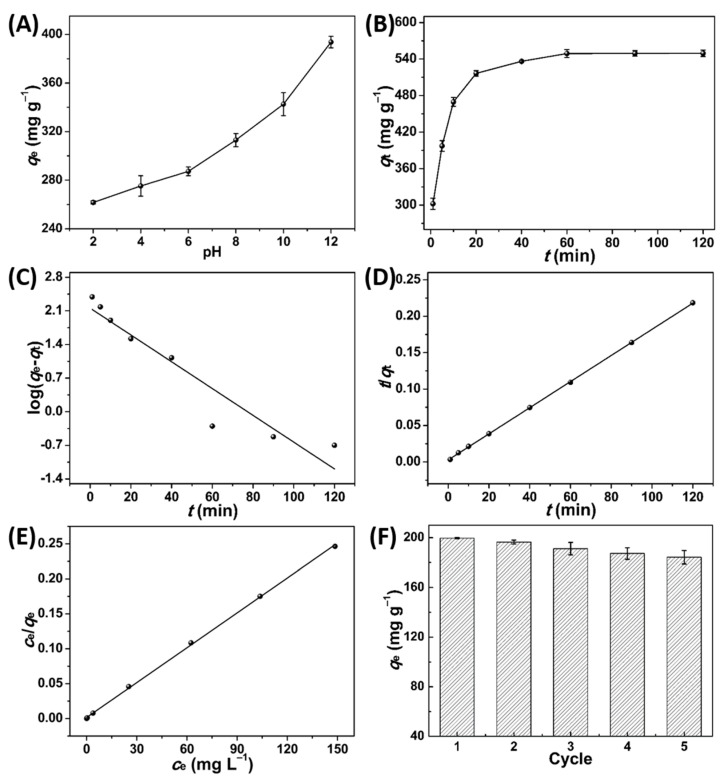
Effect of (**A**) pH (*c*_0_: 200 g L^−1^, 2 h, 25 °C); (**B**) *t* (*c*_0_: 200 g L^−1^, pH: 12, 25 °C); (**C**) pseudo-first order kinetic; (**D**) pseudo-second order kinetic; (**E**) Langmuir isotherm of MB adsorption on PPPC; and (**F**) the reusability of PPPC toward MB adsorption (*c*_0_: 100 g L^−1^, pH: 12).

For MB dye adsorption, pH is an important influencing factor [24]. It was seen that the *q*_e_ increased from 261.7 mg g^−1^ to 393.6 mg g^−1^ as pH raised from 2 to 12 (Figure 4A). According to the result, the surface charge of PPPC was negatively charged above pH 3.2 (pH_pzc_ = 3.2, Figure S1A), owed to the surface functional groups including hydroxyl and carboxyl [25], and MB is positively charged [26]. With the pH rise, the enhanced electrostatic interaction between PPPC and MB resulted in a higher *q*_e_ [12]. In addition, *q*_e_ improved with the increased *c*_0_ (Figure S1B), suggesting higher *c*_0_ could provide a stronger driving force to promote the mass transfer of MB [27]. Moreover, *q*_e_ increased with the raised adsorption temperature (Figure S1C), suggesting the adsorption process was endothermic [28]. Figure 4B showed that the adsorption was fast (302.2 mg g^−1^ min^−1^) within 1 min because of the abundant vacant adsorption sites. Then, the rate dropped, owing to the repulsive force between adsorbed dye molecules on PPPC and free dye molecules in aqueous solution [29]. Finally, the adsorption system was at equilibrium at 60 min with *q*_e_ of 549.6 mg g^−1^. It can be found that the adsorption was a rapid process, which was beneficial for the practical application of PPPC.

The pseudo-first-order kinetic and pseudo-second-order kinetic were applied to analyze the equilibrium kinetic data (Figure 4C,D; Table 1). It was shown that *R*^2^ (0.9999) of the pseudo-second order kinetic was higher than *R*^2^ (0.8973) of the pseudo-first order kinetic, and its calculated equilibrium adsorption capacity (*q*_e,cal_ = 558.7 mg g^−1^) was consistent with the experimental equilibrium adsorption capacity (*q*_e,exp_ = 549.6 mg g^−1^). This suggested that the adsorption process was well depicted by the pseudo-second order kinetic and involved the chemisorption [30]. To quantitatively study the maximum adsorption capacity (*q*_m_) and the characteristics of adsorption of MB by PPPC, Langmuir and Freundlich isotherms were employed. As shown in Figure 4E and Figure S1D, and Table 2, *R*^2^ (0.9995) of Langmuir isotherm was much higher than *R*^2^ (0.8327) of Freundlich isotherm, indicating the adsorption process obeyed Langmuir isotherm [31]. This revealed that the adsorption of MB by PPPC was mainly a monolayer adsorption, which was consistent with the previously reported porous carbons with similar pore structures [12,32,33]. Based on Langmuir isotherm, the calculated *q*_m_ was 602.4 mg g^−1^, which was larger than those of various adsorbents (Table S2). In addition, the recyclability of PPPC is crucial for its application value. Figure 4F showed that *q*_e_ only reduced by 7.7% after five cycles, suggesting that PPPC had good reusability for removing MB dye. According to our previous report [12], the stable structure of PPPC might be the main reason for its good reusability.

The possible adsorption mechanism of PPPC toward MB was summarized. Adsorption is a complicated physicochemical phenomenon related to interphase mass-transfer, surface interactions, intraparticle diffusion, and so on [34]. The excellent adsorption ability of PPPC was attributed to its abundant pores, which could reduce hindrance to accelerate the mass transfer process and capture more MB molecules [12]. Meanwhile, the electrostatic interaction and H-bonding interaction between PPPC and MB caused by the rich function groups of PPPC could influence the adsorption performance [3,10,35]. Furthermore, the π-π interaction generated by the aromatic structures of PPPC and MB was beneficial for MB adsorption [10]. From the analysis of pseudo-second order kinetic and the desorbed MB amount (Figure S2), it can be inferred that chemisorption also existed in the adsorption process. In summary, the adsorption mechanism was due to the combined effect of physisorption and chemisorption.

## 4. Conclusions

Pomelo peel-derived porous carbon with large specific surface area and abundant pore structures were prepared by a facile one-step method. The porous carbon had high adsorption capacity and excellent reusability toward MB, and the adsorption process obeyed Langmuir and pseudo-second order kinetic models. The work opened a new approach for pomelo peel utilization and a potential absorbent for wastewater treatment.

## Figures and Tables

**Table 1:** Pseudo-first order and pseudo-second order kinetics parameters.

Pseudo-First-Order Kinetics					Pseudo-Second-Order Kinetics		
*c*_0_	*q*_e,exp_	*q*_e,cal_	*k*_1_	*R* ^2^	*q*_e,cal_	*k*_2_	*R* ^2^
(mg L^−1^)	(mg g^−1^)	(mg g^−1^)	(min^−1^)		(mg g^−1^)	(g mg^−1^ min^−1^)	
300	549.6	143.4	0.06	0.8973	558.7	0.0012	0.9999

**Table 2:** Langmuir and Freundlich isotherms parameters.

Langmuir			Freundlich		
*q*_m_ (mg g^−1^)	*b* (L mg^−1^)	*R* ^2^	*k*	1/*n*	*R* ^2^
602.4	1.02	0.9995	298.33	0.17	0.8327

## Data Availability

Not applicable.

## Supplementary Materials

The following are available online at https://www.mdpi.com/article/10.3390/molecules27103096/s1. Figure S1: (A) Zeta potential of PPPC at different pH. Effect of (B) *c*0 and (C) *T* for MB adsorption on PPPC and (D) Freundlich isotherm for MB adsorption on PPPC. Figure S2: The desorbed MB concentrations in ethanol for five cycles. Table S1: The specific surface area of various porous carbon materials. Table S2: The *q*_m_ of various adsorbents [3,6,17,18,36,37,38,39,40,41,42,43,44,45,46,47].
