# Supplementary material for: Facile Preparation of Porous Carbon Derived from Pomelo Peel for Efficient Adsorption of Methylene Blue
Source: Molecules. 2022 May 11;27(10):3096. doi: 10.3390/molecules27103096 (PMC9144290; doi:10.3390/molecules27103096)
Supplement: Supplementary file 1 [file molecules-27-03096-s001.zip › molecules-1711921-supplementary.pdf]

Supplementary Materials

Facile Preparation of Porous Carbon Derived from Pomelo Peel for Efficient adsorption of Methylene Blue

Table S1. The specific surface area of various porous carbon materials.

Porous carbon materials	S_{BET} (m^2g^{-1})	References
Graphene oxide gel derived porous carbons	378	[36]
MWNTs-loaded microporous carbons	463	[37]
Polystyrene-based hierarchical porous carbons	543	[38]
Fe/ordered mesoporous carbons	731	[39]
N and P co-functionalized three-dimensional porous carbon	743	[40]
Pomelo peels-derived porous activated carbon microsheets	807.7	[41]
Honeycomb-like porous carbon derived from pomelo peel	832	[17]
Porous carbon derived from pomelo peel	1892.1	[18]
**Pomelo peel derived porous carbon**	**939.4**	**This work**

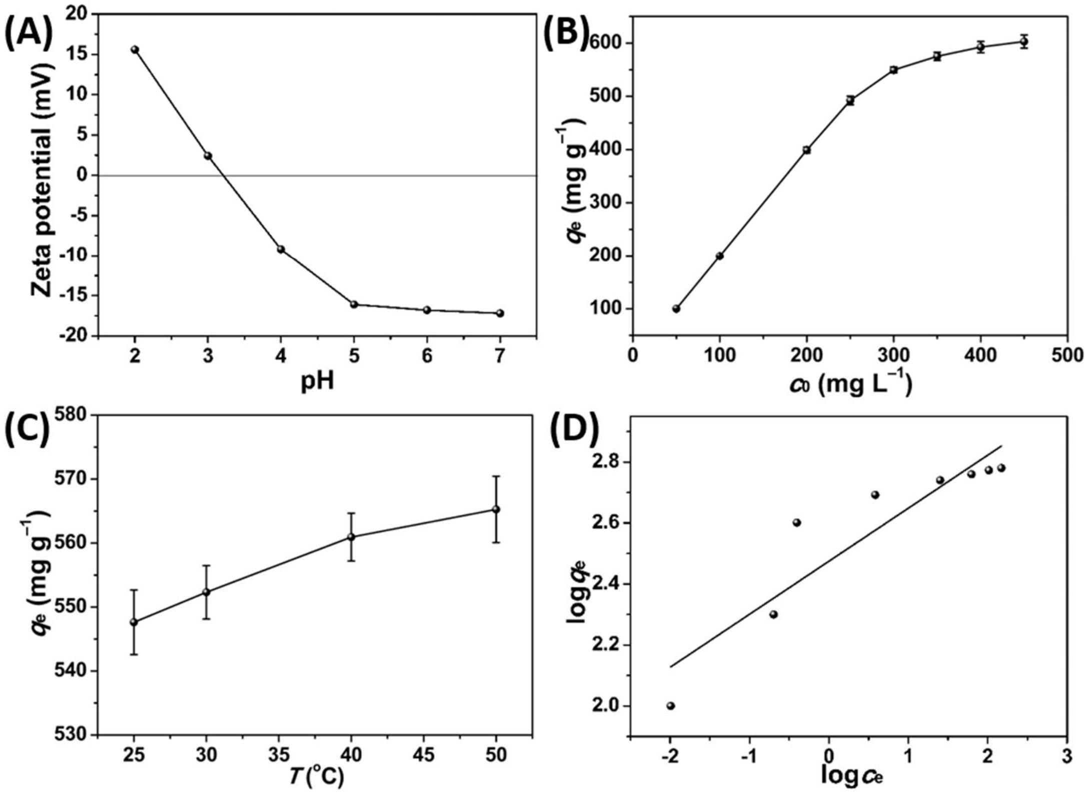

Figure S1. (A) Zeta potential of PPPC at different pH. Effect of (B) c_0 and (C) T for MB adsorption on PPPC. (D) Freundlich isotherm for MB adsorption on PPPC.

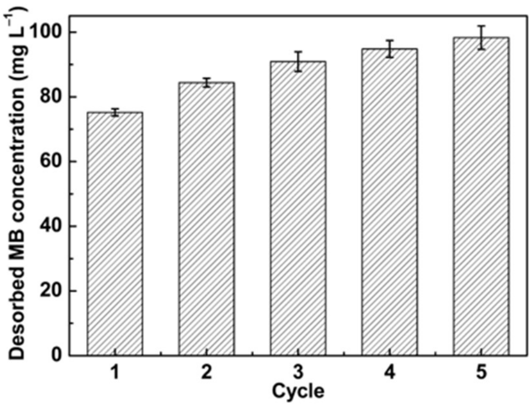

Figure S2. The desorbed MB concentrations in ethanol for five cycles.

Table S2. The q_m of various adsorbents toward MB.

Adsorbents	q_m (mg g^{-1})	References
PDA-rGO-kaolin	39.663	[6]
polyamide-vermiculite nanocomposites	76.42	[42]
Citrus pectin derived Fe_3O_4@C nanoparticles	141.3	[43]
Porous soy protein isolate based composite beads	272.4	[44]
Ginger straw derived porous carbons	345.0	[3]
Mesoporous activated carbon	359	[45]
Mesoporous magnesium silicate	382	[46]
TiO_2@C nanosheets	441	[47]
**Pomelo peel derived porous carbon**	**602.4**	**This work**
